# A Re-Audit of the Assessment and Management of Alcohol Misuse Following Admission to the General Adult Inpatient Wards in Mersey Care NHS Foundation Trust

**DOI:** 10.1192/bjo.2026.11699

**Published:** 2026-06-30

**Authors:** Joe Wilson, Ellie Rigby, Declan Hyland

**Affiliations:** 1 University of Liverpol, Liverpool, United Kingdom; 2 Mersey Care NHS Foundation Trust, Liverpool, United Kingdom

## Abstract

**Aims:**

Harmful and dependent alcohol use frequently co-occurs with psychiatric disorders and is more prevalent amongst psychiatric inpatients than in the general population. Patients admitted to general adult psychiatric wards are at increased risk of alcohol withdrawal.Early identification of alcohol misuse at admission is essential to guide appropriate investigation, monitoring, pharmacological management, and referral to specialist alcohol services.

This re-audit of assessment and management of alcohol misuse in a general adult psychiatric inpatient setting in Mersey Care NHS Foundation Trust follows the original audit conducted in 2021 and re-audit in 2022 and evaluated whether assessment and management of harmful or dependent alcohol use, as defined by ICD-11 criteria, had improved since previous audits and assess effectiveness of previous recommendations implemented.Key areas reviewed were documentation of alcohol history, identification of alcohol misuse, monitoring for withdrawal, prescription of evidence-based treatments, appropriate investigations, and referral to community alcohol services.

**Methods:**

A retrospective audit of all patients on eight general adult inpatient wards in the Trust was conducted on 22^nd^of April 2025. The electronic patient record and prescription chart for each patient were reviewed. Patients with no documented history of alcohol misuse were excluded from monitoring for and management of withdrawal. Audit standards were based on the Trust’s alcohol detoxification policy and NICE guidance.

**Results:**

The total sample was 130 inpatients - 52% male, 48% female.55% of patients had an alcohol history documented on admission.28% of patients had a prior history of alcohol misuse but only 28% of these had their average weekly alcohol intake recorded.Among patients with identified alcohol misuse, a CIWA score was documented within 24 hours in 17% - an improvement from 1% in 2021 and 4% in 2022. Prescribing of withdrawal-related medications remained inconsistent - 14% were initiated on Chlordiazepoxide, 25% on Thiamine, and 3% on Vitamin B Compound Strong within 24 hours of admission.Relevantblood tests - gamma GT level - 31% and Mg2+ - 25% were incompletely performed. No patients were offered referral to community alcohol services, a deterioration compared with previous audits.

**Conclusion:**

Despite modest improvements in CIWA documentation, significant gaps remain in assessment and management of alcohol misuse in general adult inpatients.Inconsistent clerking practices, limited use of structured proformas, and poor referral rates to community alcohol services represent missed opportunities to reduce withdrawal-related morbidity and support long-term recovery.Increased use of clerking proformas and targeted education on alcohol withdrawal management were recommended.

